# The Potential of Metabolism-Related Gene OGDHL as a Biomarker for Myocardial Remodeling in Dilated Cardiomyopathy

**DOI:** 10.3389/fcvm.2022.741920

**Published:** 2022-04-07

**Authors:** Yaohan Tang, Yaoxi Zhu, Yang Lu, Hongmin Yang, Han Yang, Lixia Li, Changhu Liu, Yimei Du, Jing Yuan

**Affiliations:** Department of Cardiology, Union Hospital, Tongji Medical College, Huazhong University of Science and Technology, Wuhan, China

**Keywords:** OGDHL, biomarker, myocardial remodeling, metabolism-related genes, dilated cardiomyopathy, fibrosis, bioinformatics

## Abstract

The development of dilated cardiomyopathy (DCM) is accompanied by a series of metabolic disorders, resulting in myocardial remodeling or exacerbation, while the mechanism remains not completely clear. This study was to find out the key metabolism-related genes involved in the onset of DCM, providing new insight into the pathogenesis of this disease. The datasets of GSE57338, GSE116250, and GSE5406 associated with hearts of patients with DCM were downloaded from the Gene Expression Omnibus database. GSE57338 was analyzed to screen out metabolism-related differentially expressed genes (DEGs), while GSE116250 and GSE5406 were utilized to verify the optimal genes through R software. Support vector machine recursive feature elimination algorithm and least absolute shrinkage and selection operator algorithm were used to determine key genes. Finally, 6 of 39 metabolism-related DEGs were screened out and identified as the optimal genes. After quantitative reverse-transcription polymerase chain reaction (qRT-PCR) validation performed on the samples drawn from the left ventricles of human hearts, it showed that only the expression of oxoglutarate dehydrogenase-like (OGDHL) increased while PLA2G2 decreased significantly in patients with DCM compared with non-failing donors, respectively. Furthermore, the higher OGDHL protein expression, except the change of PLA2G2, was also found in DCM hearts, and its mRNA expression was negatively correlated with myocardial Masson’s scores (*r* = –0.84, *P* = 0.009) and left ventricular end-diastolic diameter (LVEDd; *r* = –0.82, *P* = 0.014), which might be regulated by miR-3925-5p through further bioinformatics prediction and qRT-PCR verification. The data then suggested that the metabolism-related gene OGDHL was associated with myocardial fibrosis of DCM and probably a biomarker for myocardial remodeling in patients with DCM.

## Introduction

Dilated cardiomyopathy (DCM) is currently the common clinical cause of heart failure (HF) and heart transplantation, with the characterization of enlarged left or both ventricles and myocardial systolic dysfunction (1). It has been reported that, nowadays, there are an estimated 62 million patients with DCM worldwide, and the 5-year mortality rate is about as high as 50%, seriously threatening human health (2). As the etiology of DCM is complex, including family inheritance, gene mutation, autoimmune abnormalities, viral infection, and so on (1), the molecular mechanism and functional genes of DCM are still unclear, which has been a bottleneck problem for early diagnosis and treatment of this disease.

Besides the abnormal activation of the neuroendocrine, in 2004, Bilsen proposed that pathological cardiac hypertrophy or HF is accompanied by disorders in myocardial energy and substrate metabolism (3). In recent years, it was supported by growing experimental and clinical evidence. For example, the overexpression of calcium/calmodulin-dependent protein kinase II (CaMKII) could decrease ATP concentration by reducing the assembled complex I and the mitochondrial isoform of creatine kinase, leading to DCM in mice (4). The inhibition of angiopoietin-like protein 2 (ANGPTL2) would activate AKT-SERCA2a signaling and enhance energy metabolism in human cardiomyocytes, which blocks HF development (5). Moreover, several clinical studies have shown that the metabolism-related drugs, such as trimetazidine (6), coenzyme Q10 (7), and particular sodium-glucose cotransporter 2 inhibitors (SGLT2i) (8), present the beneficial effects on the improvement of prognosis in patients with HF as adjuvant therapies by regulating the metabolic imbalance of cardiac myocytes. Although it has been indicated as a new approach to the diagnosis and treatment of HF, the molecular mechanism of metabolism-related genes in DCM is not yet fully elucidated.

In this study, we aimed to screen, analyze, and identify the significantly different metabolic genes and metabolic pathways between the left ventricle samples of patients with DCM and those of non-failing (NF) donors, by using gene expression microarray datasets from the Gene Expression Omnibus (GEO) database. Also, the data might further reveal the pathogenesis of DCM and provide the potential metabolic targets for DCM diagnosis and treatment.

## Materials and Methods

### Data Acquisition and Metabolism-Related Genes

The dataset GSE57338, containing 82 patients with DCM and 136 NF donors; GSE116250, containing 37 patients with DCM and 14 NF donors; and GSE5406, containing 86 patients with DCM and 16 NF donors, were downloaded from GEO.^1^ The datasets are based on the GPL11532 Affymetrix Human Gene 1.1 ST Array platform, GPL96 Affymetrix Human Genome U133A Array, and GPL570 Affymetrix Human Genome U133 Plus 2.0 Array, respectively. The annotation files for GPL570, GPL96, and GPL11532 were also downloaded from the GEO. Notably, 70 metabolic pathways were collected from the Kyoto Encyclopedia of Genes and Genomes (KEGG) database (9–11). After integrating the genes in these pathways, we obtained a total of 1,435 candidate metabolism-related genes.

### Screening of Metabolism-Related Differentially Expressed Genes and Enrichment Analysis

R software (R 4.0.2) was used to analyze the obtained files. The “oligo” (12) package and “affy” (13) package were used to read GSE57338, GSE116250, and GSE6504, and the robust multi-array average (RMA) algorithm was used for background correction and data standardization. The “limma” (14) package was used to identify metabolism-related differentially expressed genes (DEGs) between DCM and NF donors. *P-*values were adjusted by the Benjamini and Hochberg method, and then, genes with *P* < 0.05 and fold change > 1.5 were considered as metabolism-related DEGs. The biological function of metabolism-related DEGs was identified by Gene Ontology (GO) and KEGG pathway enrichment analyses using an online tool WebGestalt.^2^ KEGG pathways were analyzed with Gene Set Enrichment Analysis (GSEA), using the “clusterProfiler” (15) package to define every functional cluster. C2.all.v6.2.symbols.gmt was selected as the reference gene set. False discovery rate < 0.05 and *P* < 0.05 were set as the cutoff criteria.

### Screening and Verification of Key Genes

We used least absolute shrinkage and selection operator (LASSO) regression and support vector machine recursive feature elimination (SVM-RFE) to perform feature selection to screen optimal genes for DCM. The LASSO algorithm and SVM-RFE were applied with the “glmnet” (16) package and “e1071” (17) package, respectively. Ultimately, we combined the genes from either LASSO or SVM-RFE algorithm for further analysis.

### Sample Collection

The heart tissues were taken from Union Hospital of Tongji Medical College, Huazhong University of Science and Technology. Left ventricular free wall tissues were collected from patients with transplanted DCM (*n* = 8) and NF donors (*n* = 4) and then quickly frozen with liquid nitrogen. All the heart tissues were not taken out of liquid nitrogen until use. It should be noted that this research was conducted in accordance with the purpose of the Declaration of Helsinki and its amendments and approved by the Ethics Committee of Union Hospital Affiliated of Tongji Medical College of Huazhong University of Science and Technology (Number: UHCT21001). The patients/participants provided a signed informed consent form. The clinical data of the 8 patients with DCM are shown in Table 1.

**TABLE 1:** Clinical characteristics of 8 dilated cardiomyopathy (DCM) patients before heart transplantation.

	DCM1	DCM2	DCM3	DCM4	DCM5	DCM6	DCM7	DCM8
Age (years)	59	15	52	47	62	47	20	52
Male	+	+	+	+	+	+	+	+
NYHA classification	IV	IV	IV	IV	IV	IV	IV	IV
DCM family history	–	–	–	–	–	–	–	–
VMC history	–	–	–	+	–	–	+	–
Smoke	+	–	+	+	–	+	–	–
Drink	+	–	+	+	–	+	+	+
Disease course (years)	8	1.1	5.2	11.1	7.1	4.5	1.2	6.2
SBp (mmHg)	126	100	92	110	91	94	105	122
DBp (mmHg)	72	59	58	69	51	79	55	78
Heart rate (bpm)	56	107	67	80	70	90	78	84
Atrial fibrillation	–	+	–	–	–	–	–	+
ALT (U/L)	55	45	15	153	24	17	14	14
AST (U/L)	35	17	24	43	18	19	19	10
Cr (umol/L)	114.6	107	86.1	83.3	66.7	93	92.3	89.5
LVEF (%)	13.6	14.2	22.5	12.6	11.5	15	15	15.2
LVEDd (cm)	7.9	3.9	6.4	9	7.8	7.6	6.7	8.3
NT-proBNP (pg/ml)	733	491	5300	1076	2759	2450	9481	1164
OGDHL mRNA levels	4.387	7.349	7.593	1.078	4.177	4.582	3.690	3.622

*DCM, dilated cardiomyopathy; NYHA, New York Heart Association; VMC, viral myocarditis; ALT, alanine aminotransferase; AST, aspartate aminotransferase; Cr, creatinine; LVEF, Left ventricular eject fraction; LVEDd, left ventricular end-diastolic diameter; NT-proBNP, N-terminal fragment of the BNP precursor; BNP, Brain Natriuretic Peptide.*

### Quantitative Reverse-Transcription PCR

Cardiac miRNA and mRNA expressions were measured by quantitative reverse-transcription PCR (qRT-PCR). The total RNA of heart tissues was extracted using the TRIzol reagent (Takara, Otsu, Japan) according to the protocol, then using kits (Takara, Otsu, Japan) to reverse transcribe RNA into cDNA. qRT-PCR was performed with primers on the real-time fluorescent quantitative PCR system (Bio-Rad, Hercules, CA, United States). All the reactions were conducted at least in duplicate for each sample. The level of gene expression was calculated by the 2^–Δ^ ^Δ^ *^Ct^* method. The reverse primer was the universal primer in the kit (GeneCopoeia, Rockville, MD, United States). The sequences of other primer pairs are presented in Table 2.

**TABLE 2:** The sequence of primers.

Primer name	Sequence
GAPDH-F	5′- ACAACTTTGGTATCGTGGAAGG-3′
GAPDH-R	5′- GCCATCACGCCACAGTTTC-3′
OGDHL-F	5′-GGGCGTGGTATATGAGACCTT-3′
OGDHL-R	5′-TGTGGTGAATCCAATCTGGTTG-3′
PLA2G2A-F	5′-ATGAAGACCCTCCTACTGTTGG-3′
PLA2G2A-R	5′-GCTTCCTTTCCTGTCGTCAACT-3′
HMOX2-F	5′-TCAGCGGAAGTGGAAACCTC-3′
HMOX2-R	5′-AGAAGTCCTTGACAAACTGGGT-3′
ANPEP-F	5′-TTCAACATCACGCTTATCCACC-3′
ANPEP-R	5′-AGTCGAACTCACTGACAATGAAG-3′
CHDH-F	5′-GGCTGGCTCAAACTGAGAAGT-3′
CHDH-R	5′-CACACAGACGGAAATCCTCAAT-3′
ALG3-F	5′-CCGAGGTAGAAGGCGTCATC-3′
ALG3-R	5′-GGTACACAAGTGGTCCGGT-3′
U6-F	5′- GCTTCGGCAGCACATATACTAAAAT-3′
miR-3925-5p	5′- CGCCGAAGAGAACTGAAAGTGGAGCC-3′

### Western Blot

Total proteins of the heart tissues were extracted using the Total Protein Extraction Kit (Pierce/Thermo Scientific Waltham, MA, United States). The protein concentration was determined using the BCA Protein Assay Kit (Pierce). Samples containing 40 μg proteins were separated on a 10% SDS–PAGE and electro-transferred onto nitrocellulose membranes. Membranes were blocked for 2 h in Tris-buffered saline (pH 7.6)–0.2% Tween (TBST) containing 5% skim milk and incubated with primary antibodies against oxoglutarate dehydrogenase-like (OGDHL) (1:500, Abcam, ab100928) and GAPDH (1:3000, Abcam, ab9485) overnight at 4°C. After washing, the membranes were incubated with horseradish peroxidase (HRP)-conjugated secondary antibodies (1:3000) at 37°C for 2 h. The target bands were finally developed with super ECL reagent (Thermo Scientific, United States), captured by Image Lab, and semiquantitatively analyzed with densitometric methods.

### Histology

The hearts were embedded in the optimal cutting temperature compound and processed for cryosections at 12 μm. For immunohistochemistry, cryosections were treated with 3% H_2_O_2_ for 10 min. After washing with phosphate-buffered saline (PBS) 3 times and blocking with 3% bovine serum albumin (BSA) for 30 min, the sections were incubated with OGDHL (1:50, Abcam, ab100928) at 4°C overnight and washed with PBS 3 times. After incubation with HRP-conjugated anti-rabbit antibody for 45 min and washing adequately, diaminobenzidine solution was added, and the sections were counterstained by hematoxylin. For Masson’s trichrome staining, the cryosections were stained with Masson dye. Images were visualized, and digital photographs were taken using a Zeiss microscope.

### Correlation Analysis

Areas of fibrosis relative to the total left ventricular area were measured and expressed as percentages by computerized planimetry using Image-Pro Plus 6.0 software. Five images per heart were analyzed to obtain mean values. Spearman’s correlation analysis was performed for correlation between gene expression and fibrosis area.

### Statistical Analysis

Unless specifically noted, all quantitative data are presented as mean ± SEM. Statistical analysis was performed by test using GraphPad Prism 8.0, and *P* < 0.05 was considered statistically significant.

## Results

### Identification of Metabolism-Related Differentially Expressed Genes From Patients With Dilated Cardiomyopathy

**FIGURE 1:**
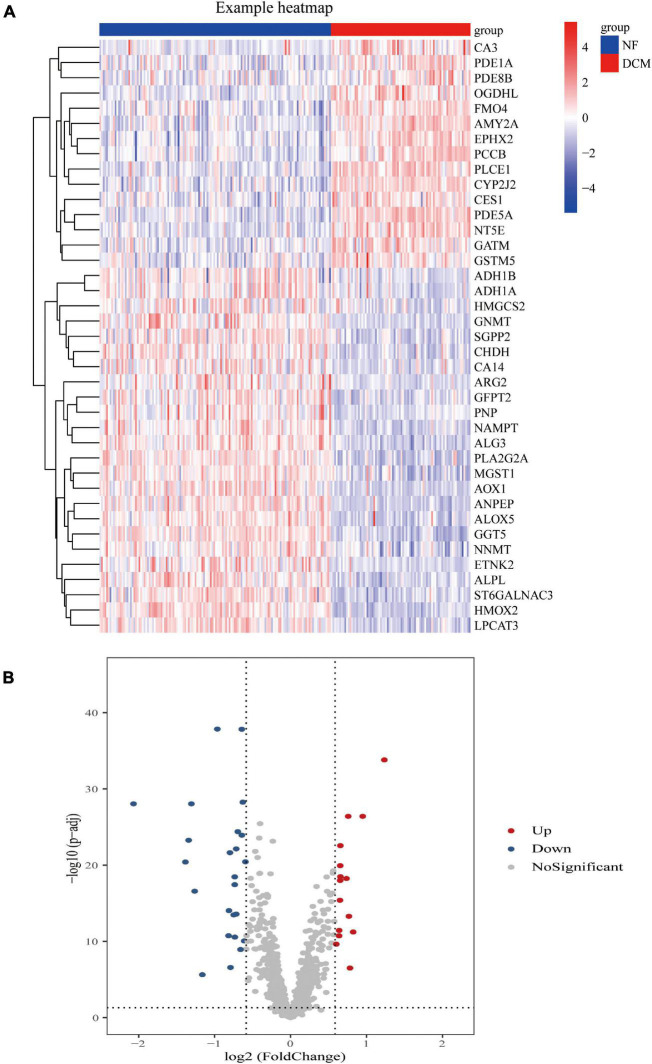
Differential expression of feature metabolism-related genes in dilated cardiomyopathy (DCM) patients and non-failing (NF) donors. Heat map **(A)** and volcano map **(B)** of metabolic-related differential gene expression in left ventricular tissues of patients with DCM and NF donors.

Compared with the samples from the NF hearts, a total of 39 metabolism-related DEGs, containing 15 upregulated and 24 downregulated genes, were identified from DCM hearts. Based on the difference in gene expression, these 39 metabolism-related DEGs were shown in a heat map (Figure 1A) and in a volcano plot (Figure 1B).

### Functional and Pathway Enrichment Analysis

**FIGURE 2:**
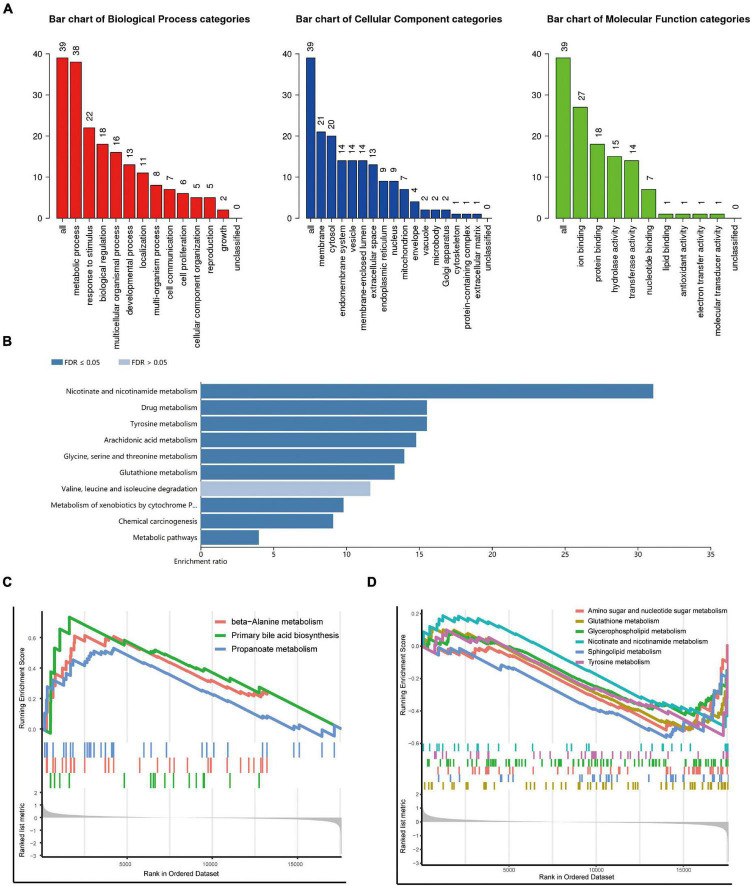
Functional and pathway enrichment analysis. **(A,B)** Gene Ontology (GO) **(A)** and Kyoto Encyclopedia of Genes and Genomes (KEGG) **(B)** enrichment analysis of the metabolism-related genes. **(C,D)** Representative Gene Set Enrichment Analysis (GSEA) results of the upregulated **(C)** and downregulated **(D)** metabolism-related pathways.

We conducted a functional enrichment analysis to further study DEGs. As shown in Figure 2A, GO analysis results presented that the biological process terms of these 39 genes were significantly enriched in metabolic process, response to stimulus, and biological regulation. Meanwhile, the location was mainly membrane and cytosol, and molecular function terms were significantly enriched in ion binding, protein binding, hydrolase, and transferase activity. KEGG enrichment showed several biological pathways, including nicotinate and nicotinamide metabolism, drug metabolism, and tyrosine metabolism (Figure 2B).

To identify changes in metabolic pathways at the overall gene expression level, we performed GSEA. The GSEA revealed that primary bile acid biosynthesis and beta-alanine metabolism were upregulated in patients with DCM (Figure 2C), while glutathione metabolism, sphingolipid metabolism, amino sugar, and nucleotide sugar metabolism were downregulated (Figure 2D).

### Screening and Verification of Key Genes

**FIGURE 3:**
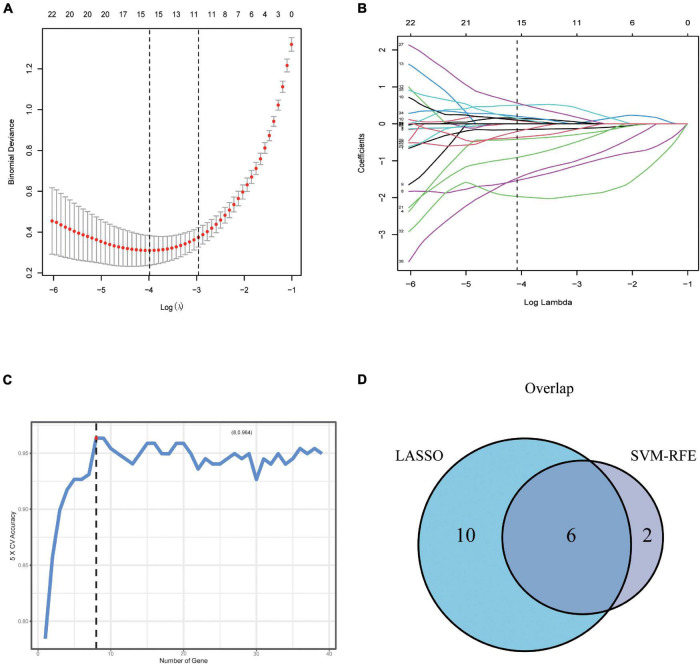
Screening and verification of key genes. **(A,B)** Least absolute shrinkage and selection operator (LASSO) logistic regression algorithm to screen candidate feature genes. **(C)** Support vector machine recursive feature elimination (SVM-RFE) algorithm to screen candidate genes. **(D)** The Venn diagram shows the intersection of diagnostic markers obtained by the two algorithms.

**FIGURE 4:**
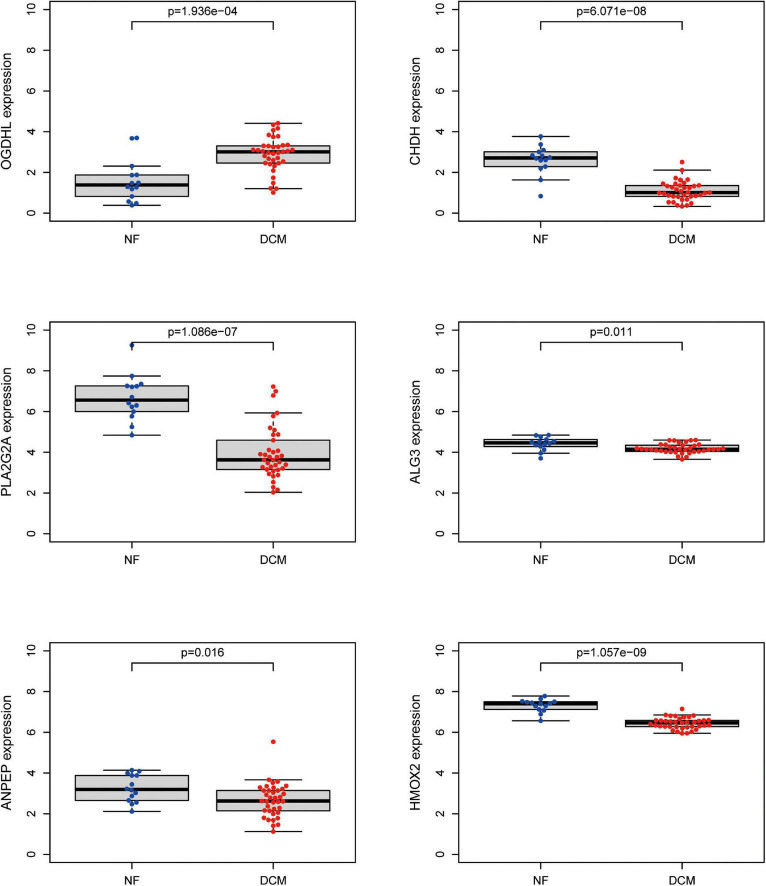
Validation of expression level of key genes. The expression of the six key metabolism-related genes in the GSE116250 dataset.

To further screen optimal genes, we analyzed DEGs by LASSO regression and SVM-RFE. The R software packages “glmnet” and “e1071” were used for LASSO regression and SVM-RFE, respectively. The LASSO regression algorithm identified 16 genes from 39 metabolism-related DEGs in DCM and NF samples (Figures 3A,B). The SVM-RFE algorithm was used to identify 8 characteristic genes from 39 metabolism-related DEGs *via* fivefold cross-validation (Figure 3C). Finally, six overlapping genes, namely, PLA2G2A, ANPEP, CHDH, OGDHL, HMOX2, and ALG3, were extracted (Figure 3D) and then verified in GSE116250 and GSE5406. As shown in Figure 4 and Supplementary Figure 1, the expression trend of these genes (except CHDH in GSE5406) is consistent with this dataset.

### The Validation of Key Gene Expression in Human Hearts

**FIGURE 5:**
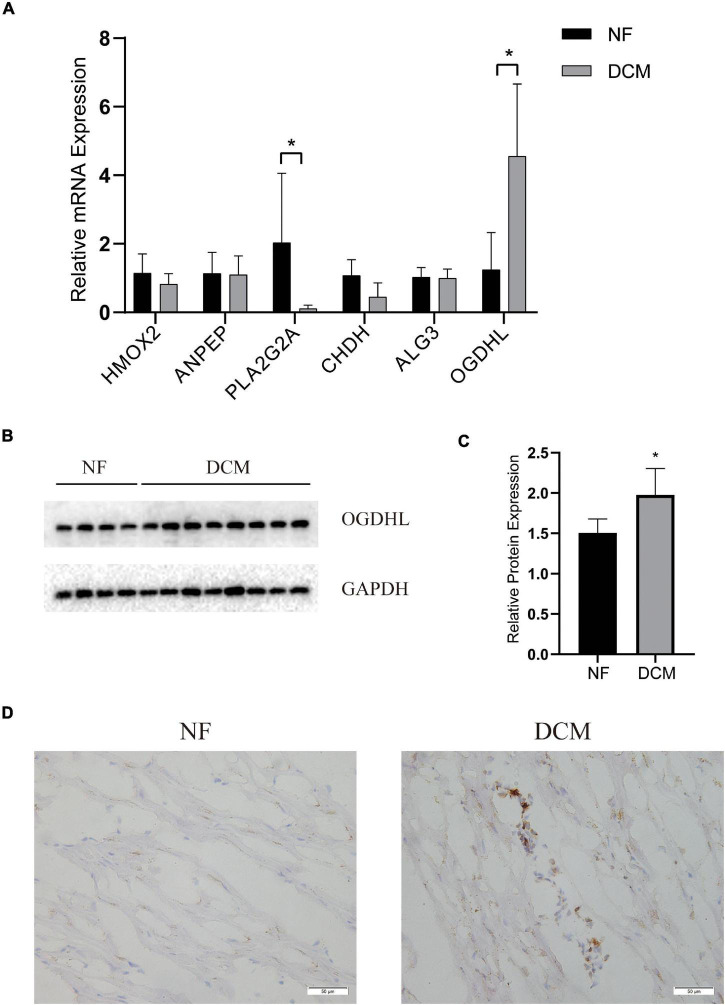
The validation of key genes expression in human hearts. **(A)** The mRNA levels of the key genes expression and **(B,C)** the protein levels of OGDHL expression in left ventricular tissues of patients with DCM and NF donors. **(D)** Representative OGDHL immunohistochemistry images of heart sections from patients with DCM and NF donors (magnification 400×). *P < 0.05 vs. NF. *n* = 4–8/group.

To further verify which metabolism-related genes might play a significant role in the progression of DCM, the mRNA levels of PLA2G2A, ANPEP, CHDH, OGDHL, HMOX2, and ALG3 were determined by using qRT-PCR. We noted that OGDHL was upregulated while PLA2G2A was downregulated significantly in DCM heart tissues compared with NF heart tissues, which is in accordance with the results of the abovementioned bioinformatics analysis (Figure 5A). Moreover, the higher OGDHL protein expression, except the change of PLA2G2, was also found in DCM hearts (Figures 5B–D), further indicating the importance of OGDHL in this disease.

### OGDHL Expression Is Negatively Correlated With Heart Fibrosis in Patients With Dilated Cardiomyopathy

**FIGURE 6:**
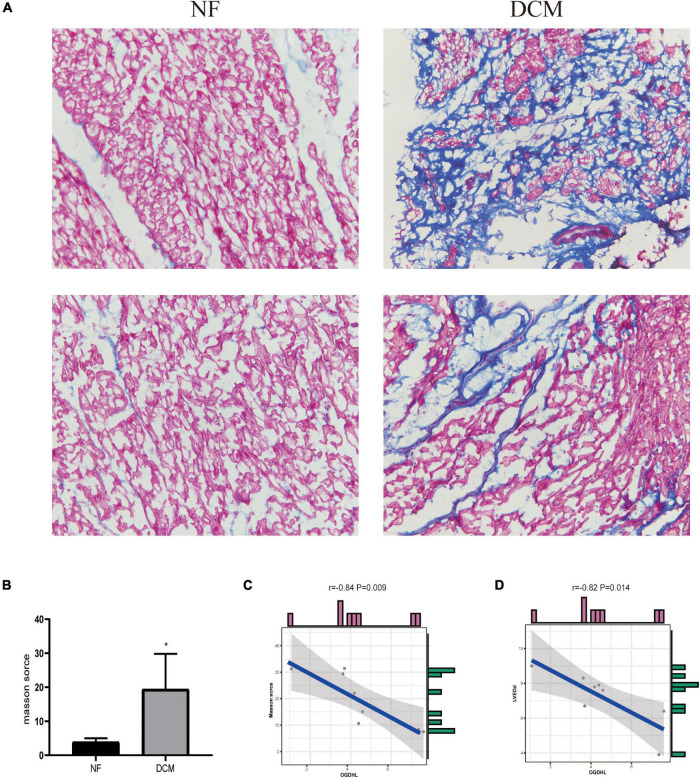
OGDHL expression is negatively correlated with heart fibrosis in patients with DCM. **(A)** Representative Masson’s stained images of heart sections from patients with DCM and NF donors (magnification 200×). **(B)** Masson’s staining scores of DCM group and NF group. **(C)** Correlation analysis between Masson’s score and OGDHL mRNA level. **(D)** Correlation analysis between OGDHL mRNA level and left ventricular end-diastolic diameter (LVEDd). *P < 0.05 vs. NF. *n* = 4–8/group.

Regarding that the myocardial fibrosis is the basic pathophysiological change of DCM, we analyzed the correlation between the fibrosis area detected by Masson’s trichrome staining and the OGDHL expression detected by qRT-PCR and found that the expression of OGDHL at mRNA level was negatively correlated with the fibrosis area (*r* = –0.84, *P* = 0.009) (Figures 6A–C).

### OGDHL Expression Is Negatively Correlated With Left Ventricular End-Diastolic Diameter in Patients With Dilated Cardiomyopathy

After analyzing the clinical characteristics of those 8 patients with DCM who underwent heart transplantation as shown in Table 1, we found that OGDHL mRNA expression levels were also negatively correlated with LVEDd (*r* = –0.82, *P* = 0.014) (Figure 6D).

### Identification of Candidate OGDHL-Targeting miRNAs

**FIGURE 7:**
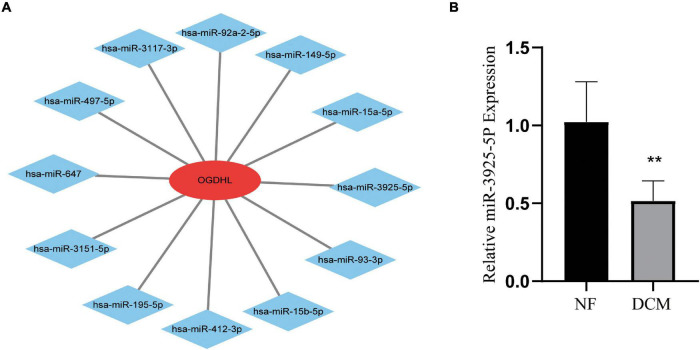
Identification of candidate OGDHL-targeting miRNA. **(A)** The predicted 12 candidate OGDHL-targeting miRNAs. **(B)** The expression level of miR-3925-5p in left ventricular tissues of patients with DCM and NF donors. **P < 0.01 vs. NF. *n* = 3–8/group.

Regarding OGDHL might play an important role in DCM, we then use the miRWalk 2.0 software to predict OGDHL-targeting miRNAs. Also, it showed that a total of 12 miRNAs were predicted as targets by four algorithms (i.e., miRWalk, miRanda, RNA22, and TargetScan) using the miRWalk database (Figure 7A). After validating these 12 miRNAs in heart tissues by qRT-PCR, we found that the expression level of miR-3925-5p in the heart tissues of patients with DCM was significantly lower than those in normal heart tissues, which was contrary to the results of OGDHL, with no changes in other miRNAs (Figure 7B).

## Discussion

The DCM is a heterogeneous group of myocardial diseases (18). The diagnosis of DCM is still performed by measuring the size of the left ventricle and the left ventricular ejection fraction through echocardiography (19). The specific treatment of early diagnosis of DCM needs to be further studied. Recent studies have found that metabolic disorders play an important role in DCM (20), and many miRNA biomarkers with diagnostic and therapeutic significance for DCM have also been found (21). However, there is no research on genetic biomarkers related to metabolism in DCM. In this study, we used the bioinformatics method for the first time to analyze metabolism-related DEGs of patients with DCM and NF donors in hearts and to explore their roles in DCM. Through machine learning algorithm and verification in human heart tissues, we finally determined that OGDHL is highly expressed in DCM and negatively correlated with fibrosis, providing a potential value as a biomarker for the diagnosis and treatment of DCM.

After analyzing the datasets downloaded from GEO, 39 metabolism-related DEGs were screened, including 15 upregulated and 24 downregulated genes. The GO enrichment analysis showed that these DEGs were mainly related to metabolic processes, response to stimulus, and biological regulation. The KEGG enrichment analysis showed that DEGs were mainly related to nicotinate and nicotinamide metabolism, drug metabolism, and tyrosine metabolism. Consistently, nicotinamide riboside has been reported to preserve cardiac function in a mouse model of DCM (22, 23). In addition, the pathways identified by GSEA mainly include primary bile acid biosynthesis, beta-alanine metabolism, and glutathione metabolism.

By combining SVM-RFE and LASSO regression methods, six key genes were screened out. After validation in human heart tissues, we found that OGDHL expression significantly increased at mRNA and protein levels. OGDHL is a subunit of α-ketoglutarate dehydrogenase complex (α-KGDHC) and is also one of its rate-limiting components (24). It participates in the tricarboxylic acid cycle and regulates the metabolism of sugar and glutamate in tissues and cells (24). A preliminary study of OGDHL has found that its protein is enriched in the brain but not in the heart (25). Subsequent experiments are mainly focused on cancers, for example, it could inhibit the growth of liver cancer cells (26) but promoted pancreatic cancer metastasis (27). Recently, however, it was reported that OGDHL missense mutation can lead to mitochondrial dysfunction, and knockout of Zbtb20 in mice directly negatively regulates OGDHL generation, resulting in reduced ATP generation and decreased cardiac function (28), while its cardio-specific overexpression can improve mitochondrial function and cardiac function in mice (29). In this study, based on computer prediction, we verified that the higher the OGDHL was in the myocardial tissue of patients with DCM, the lower the degree of fibrosis was. It then further implied that OGDHL may play a protective role in energy metabolism of cardiomyocytes, which consequently inhibited the myocardial remodeling of DCM.

At present, there are few studies about OGDHL in heart diseases. Lately, Dai showed that knockout of OGDHL in liver cancer cells stimulated the consumption of glutamine which is a precursor substance necessary for the synthesis of glutathione and an effective antioxidant (26). In contrast, glutathione supplementation has been demonstrated to enhance endogenous antioxidant capacity in patients with HF and acute myocardial infarction, resulting in improved patient outcomes (30). Therefore, glutathione is probably the target for OGDHL working in cardiomyocytes of DCM. Beyond that, as Sen has reported that knockdown of OGDHL in SiHa cells would lead to AKT activation and increase nuclear factor kappa B (NF-κB) translocation and DNA binding (31), both of which are also the crucial signaling pathways for the apoptosis of cardiomyocytes and myocardial fibrosis (32), it could be hypothesized that OGDHL contributes to DCM fibrosis by regulating glutathione metabolism and followed AKT/NF-κB pathways.

miRNA is an endogenous non-coding RNA molecule with a length of 18–22 nt, which targets the 3′ untranslated region (UTR) of a gene and can regulate gene expression at the post-transcriptional level to degrade or inhibit the translation of the target gene (21). Considering the important role of OGDHL in DCM, we predicted miRNAs that might regulate OGDHL on the website. After qRT-PCR verification, it was found that the expression of miR-3925-5p was reduced. At present, there is no report about miR-3925-5p-related research. Further research is needed to clarify the relationship between miR-3925-5p and OGDHL and to determine whether the miR-3925/OGDHL axis is a viable therapeutic target in patients with DCM.

## Limitations

The limitations should be noted in this study. First, the size of the study population was relatively small. Second, we only validated OGDHL expression in patients with DCM, and further functional experiments should be performed by *in vivo* and *in vitro* studies. Third, the mechanism of OGDHL in DCM needs further exploration.

## Data Availability Statement

The raw data supporting the conclusions of this article will be made available by the authors, without undue reservation.

## Ethics Statement

The studies involving human participants were reviewed and approved by Ethics Committee of Union Hospital Affiliated to Tongji Medical College of Huazhong University of Science and Technology. The patients/participants provided their written informed consent to participate in this study.

## Author Contributions

YT and JY designed the experiments and wrote the manuscript. YT, YL, and YZ analyzed the data. YT, YL, HoY, HaY, YZ, LL, and CL performed the experiments. YL and YD collected the patients. JY supervised the project and provided funding for the project. All authors reviewed the manuscript.

## Conflict of Interest

The authors declare that the research was conducted in the absence of any commercial or financial relationships that could be construed as a potential conflict of interest.

## Publisher’s Note

All claims expressed in this article are solely those of the authors and do not necessarily represent those of their affiliated organizations, or those of the publisher, the editors and the reviewers. Any product that may be evaluated in this article, or claim that may be made by its manufacturer, is not guaranteed or endorsed by the publisher.

## Footnotes

1
https://www.ncbi.nlm.nih.gov/geo/

2
http://www.webgestalt.org/

## Supplementary Material

Supplementary Figure 1 Validation of expression level of key genes. The expression of the key metabolism-related genes in the GSE5406 dataset.
