# Supplementary figures and images for: The Potential of Metabolism-Related Gene OGDHL as a Biomarker for Myocardial Remodeling in Dilated Cardiomyopathy
Source: Front Cardiovasc Med. 2022 Apr 7;9:741920. doi: 10.3389/fcvm.2022.741920 (PMC9021392; doi:10.3389/fcvm.2022.741920)

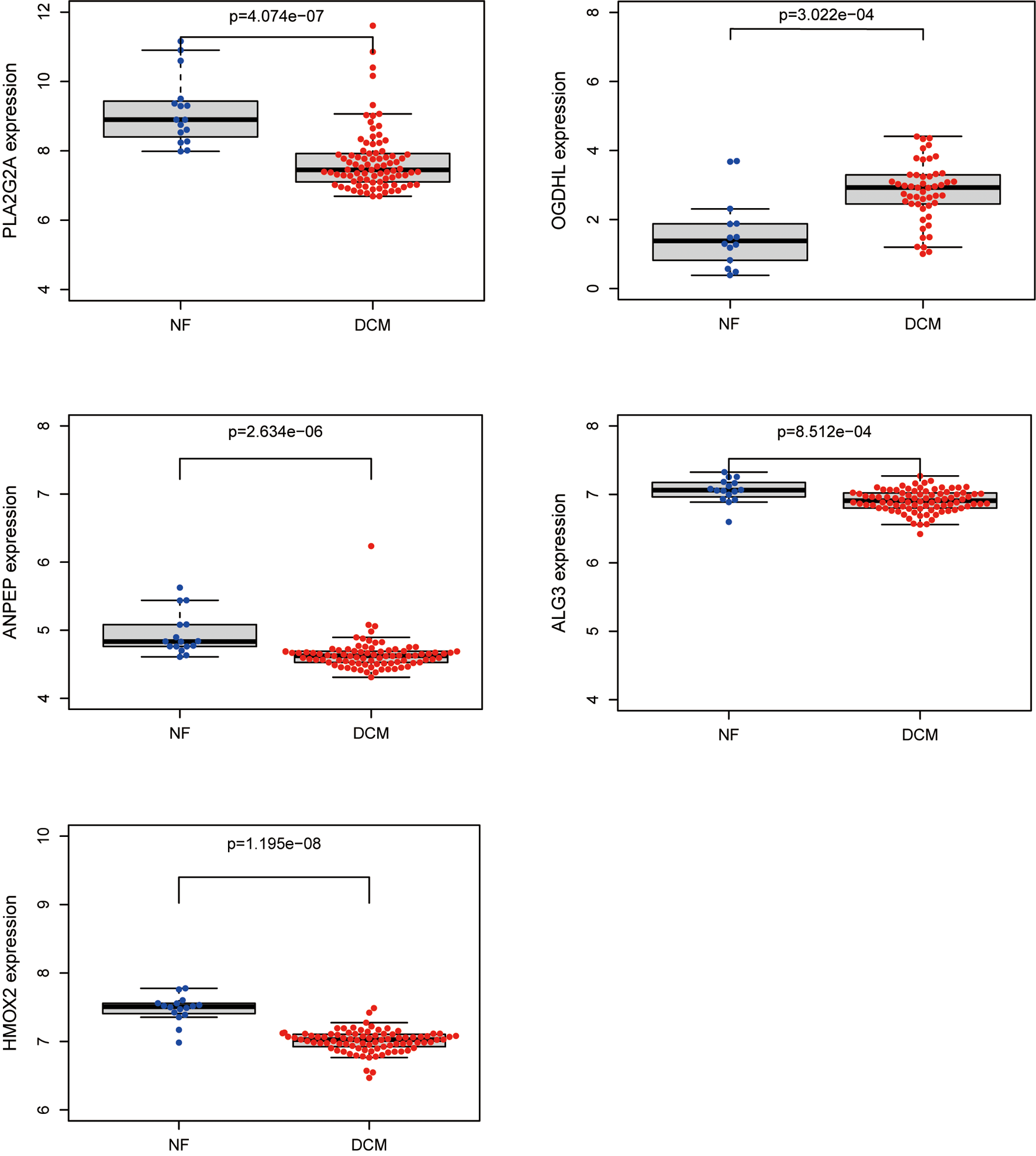

Supplement: Supplementary Figure 1 — Validation of expression level of key genes. The expression of the key metabolism-related genes in the GSE5406 dataset. [file Image_1.tif]
